# A *De Novo* Whole Genome Assembly and Annotation of *Parelaphostrongylus tenuis*

**DOI:** 10.2478/jofnem-2024-0009

**Published:** 2024-03-14

**Authors:** Tyler J. Garwood, Jessie E. Richards, Marissa G. Macchietto, Richard W. Gerhold, Stephen A. Kania, John R. Garbe, Nicholas M. Fountain-Jones, Peter A. Larsen, Tiffany M. Wolf

**Affiliations:** Department of Veterinary Population Medicine, College of Veterinary Medicine, University of Minnesota-Twin Cities, Room 495 Animal Science/Veterinary Medicine Building, 1988 Fitch Avenue, St. Paul, MN, 55108; Department of Biomedical and Diagnostic Sciences, College of Veterinary Medicine, University of Tennessee-Knoxville, 2407 River Drive, Knoxville, TN, 37996; Minnesota Supercomputing Institute, University of Minnesota-Twin Cities, 117 Pleasant Street SE, 547 Walter Library, Minneapolis, MN, 55455; Genomics Center, University of Minnesota-Twin Cities, 2231 6th Street SE, Minneapolis, MN, 55455; School of Natural Sciences, University of Tasmania, Private Bag 37, Hobart, Tasmania, Australia 7001; Department of Veterinary and Biomedical Sciences, College of Veterinary Medicine, University of Minnesota-Twin Cities, Room 239B Veterinary Science Building, 1971 Commonwealth Avenue, Saint Paul, MN, 55108

**Keywords:** Annotation, assembly, brainworm, genome, genomics, meningeal worm, *Parelaphostrongylus tenuis*, reference

## Abstract

*Parelaphostrongylus tenuis* causes ungulate morbidity and mortality in eastern and central North America, but no reference genome sequence exists to facilitate research. Here, we present a *P. tenuis* genome assembly and annotation, generated with PacBio and Illumina technologies. The assembly is 491 Mbp, with 7285 scaffolds and 185 kb N50.

## Announcement

Climate change is predicted to increase the geographic range of Protostrongylid nematodes, which cause morbidity and mortality in many wild and domestic ungulate species (Carreno and Hoberg, 1999; Kutz et al., 2005). In North America, *Parelaphostrongylus tenuis* has already expanded its range northward in the last half-century, affecting the persistence and management of wildlife and domestic species (Lankester, 2001; Pickles et al., 2013). *P. tenuis* is a driver of moose population decline in the north-central United States and south-central Canada (Lankester, 2010; Carstensen et al., 2017) and impedes translocations and reintroductions of caribou (Vors and Boyce, 2009), mule deer (Oates et al., 2000), and elk (Samuel et al., 1992). It also causes neurological symptoms and mortality in a variety of domestic species (Keane et al., 2022). Currently, there is no publicly available reference genome sequence for *P. tenuis,* or any other member of the Protostrongylidae family. Hence, the generation of a *P. tenuis* reference genome and annotation is a significant advance in the molecular study of both *P. tenuis* and Protostrongylids. Here, we present a high-quality *de novo* genome assembly and annotation of *P. tenuis*. This sequence will aid wildlife conservation and domestic animal husbandry by facilitating future studies of Protostrongylid transmission and evolution.

On October 23, 2019, we extracted two adult *P. tenuis* nematodes using methods described in Slomke et al. (1995) from a single hunter-harvested white-tailed deer doe head near Rochester, Minnesota. We determined the nematodes to be female based on their large size relative to males (Lankester, 2001). We then flash-froze the specimens in nitrogen and stored them at −80°C until DNA extraction two weeks later. We combined the two worms for a single DNA extraction, which the University of Minnesota Genomics Center (UMGC; St. Paul, MN) performed using the Gentra Puregene (Qiagen, Hilden, Germany) Tissue kit. UMGC then used the Genomic DNA ScreenTape System and Tapestation (Agilent Technologies, Santa Clara, CA) software to confirm sufficient DNA mass and quality for downstream applications. The DNA yield from the nematodes was 3.86 μg, with absorbance ratios of 1.4 at 260/230 nm and 1.85 at 260/280 nm. The DNA integrity number (scale of 1 to 10, with 1 being highly degraded and 10 being highly intact) was 8.9, and 93.11% of the fragments were between 12,198 and >60,000 bp. UMGC performed library preparation on this DNA using the PacBio SMRTbell Express Template Prep Kit 2.0 (Pacific Bioscience of California Inc., Menlo Park, CA) and carried out sequencing on a PacBio Sequel using 3 1M v3 SMRT cells.

We used PacBio SMRT^®^ Tools to create circular consensus sequences from the raw reads and perform quality filtering. We removed reads that had a minimum predicted accuracy of lower than 80%, a consensus read length of below 100 bp, or a consensus read length of above 745,000 bp. Among the approximately 1.4M reads that passed filters, the average Phred quality score was 8, and the mean length was 8,000 bp. We *de novo* assembled consensus reads with Flye (v2.7; Kolmogorov et al., 2019). Using QUAST (Gurevich et al., 2013), we generated quality statistics for our assembly. Our final assembly was 491 Mbp, with a coverage of 23X. Our assembly contained 7,285 scaffolds and had an N50 of 185 kbp (Table 1). Based on these statistics, *P. tenuis* has one of the larger genomes in the order Strongylidae (Table 2). Our assembly quality is also in the top half of publicly available genomes in Strongylidae based on N50 and number of scaffolds.

**Table 1:** Quality measures and descriptive statistics for our genome assembly and annotation of *Parelaphostrongylus tenuis*.

**Assembly/Annotation Features**	***P. tenuis* Genome**
Scaffolds (no.)	7,285
N50 (bp)	158,736
N75 (bp)	73,872
Total length (bp)	491,304,140
Longest scaffold (bp)	1,543,493
GC content (%)	40.07
Complete Genome BUSCOs (no./%)	2,818/90
Complete Genome single-copy BUSCOs (no./%)	2724/87
Complete and duplicated Genome BUSCOs (no./%)	94/3
Fragmented Genome BUSCOs (no./%)	54/1
Missing Genome BUSCOs (no./%)	259/8.3
Protein-coding genes (no.)	29,657
Non-coding tRNA genes (no.)	9,623
Complete Proteome BUSCOs (no./%)	2,224/71
Fragmented Proteome BUSCOs (no./%)	54/7.1
Missing Proteome BUSCOs (no./%)	259/21.9

**Table 2:** A comparison of descriptive statistics of the Stronglyid family genomes available in WormBase ParaSite.

**Species**	**Author/Source**	**Accession no.**	**Family**	**Number of Contigs or Scaffolds**	**Genome Size**	**N50**	**Largest Contig or Scaffold**	**GC content (%)**
Parelaphostronglus tenuis	Our genome (University of Minnesota)	GCA_019055375.1	Protostrongylidae	7,414	491.3 Mb	158.7 kb	1.5 Mb	40.1
Ancyclostoma caninum	McDonnell Genome Institute	GCA_003336725.1	Ancylostomatidae	25,335	465.7 Mb	256.7 kb	5.5 Mb	42.5
Ancylostoma ceylanicum	Cornell University	GCA_000688135.1	Ancylostomatidae	1,736	313.1 Mb	668.4 kb	4.8 Mb	43.4
Ancylostoma duodenale	McDonnell Genome Institute	GCA_000816745.1	Ancylostomatidae	100,268	332.9 Mb	10.1 kb	0.3 Mb	42.5
Angiostrongylus cantonensis	Sun Yat-sen University	GCA_009735665.1	Angiostrongylidae	35,378	293.3 Mb	860.8 kb	4.7 Mb	41.5
Angiostrongylus costaricensis	Wellcome Sanger Institute	GCA_900624975.1	Angiostrongylidae	6,384	262.8 Mb	112.5 kb	0.8 Mb	41.2
Angiostrongylus vasorum	University of Zurich	GCA_018806985.1	Angiostrongylidae	468	279.9 Mb	1,700 kb	7.3 Mb	41.6
Cylicostephanus goldi	Wellcome Sanger Institute	GCA_900617965.1	Cyathostominae	154,509	173.4 Mb	1.2 kb	0.02 Mb	40.2
Dictyocaulus viviparus	The Genome Institute	GCA_000816705.1	Dictyocaulidae	7,157	161 Mb	225.7 kb	2.2 Mb	34.5
Haemonchus contortus	Wellcome Sanger Institute	GCA_000469685.2	Trichostrongylidae	191	283.4 Mb	47,400 kb	51.8 Mb	43.1
Haemonchus placei	Wellcome Sanger Institute	GCA_900617895.1	Trichostrongylidae	24,923	259.1 Mb	37.6 kb	0.3 Mb	42.8
Heligmosomoides polygyrus	Wellcome Sanger Institute	GCA_900618505.1	Heligmosomidae	44,726	560.7 Mb	35.8 kb	0.4 Mb	42.0
Heterorhabditis bacteriophora	McDonnell Genome Institute	GCA_000223415.1	Rhabditidae	1,240	77 Mb	102 kb	2.2 Mb	33.3
Necator americanus	McDonnell Research Institute	GCF_000507365.1	Ancylostomatidae	11,864	244.1 Mb	211.9 kb	1.9 Mb	40.2
Nippostrongylus brasiliensis	Wellcome Sanger Institute	GCA_900618405.1	Heligosomidae	44,362	294.4 Mb	33.5 kb	0.4 Mb	42.7
Oesophagostomum dentatum	McDonnell Genome Institue	GCA_00079755.1	Chabertiidae	64,255	443 Mb	26.5 kb	1.6 Mb	41.4
Strongylus vulgaris	Wellcome Sanger Institute	GCA_900624965.1	Strongylidae	167,310	291.1 Mb	2.4 kb	0.09 Mb	37.9
Teladorsagia circumcincta	McDonnell Genome Institute	GCA_002352805.1	Trichostrongylidae	81,730	700.6 Mb	47.1 kb	1.5 Mb	44.5

Using BUSCO (v5.2.2; Simão et al., 2015) with the Augustus gene predictor v3.4.0 (Stanke et al., 2006), we searched our assembly for ortholog sequences from the nematode_odb10 lineage to assess genome assembly completeness. We detected 2,818 of the 3,131 BUSCO genes in our assembly, indicating that it is 90% complete, with 3% of the BUSCOs duplicated (94 BUSCO genes). Another 54 (1.7%) of BUSCO genes were fragmented, and 259 (8.3%) were missing.

To identify and annotate repetitive elements in the genome assembly, we built a custom repeat library from our assembly with RepeatModeler (v2.0.1; Smit and Hubley, 2019). We used RepeatMasker (v4.0.5; Smit et al. 2015) to combine this custom library with a standard RepeatMasker library and then ran the program in sensitive mode to find repeats in our assembly. The assembly contained 7.17% repetitive content (160,749 repeat elements, 35,213,890 bp), comprised largely of long interspersed nuclear elements (LINEs; 31,166 elements, 4.5% of genome sequence), DNA transposons (42,041 elements, 1.12% of genome sequence), long terminal repeats (LTRs; 11,592 elements, 0.16% of genome sequence), and short interspersed nuclear elements (SINEs; 3590 elements, 0.05% of genome sequence). These repetitive elements were labeled and masked with RepeatMasker so as not to interfere with later annotation steps. This amount of repeat content is low relative to other large nematode genomes, and it is possible that undermasking explains the large number of protein-coding genes predicted.

We used RNA sequencing data to inform the gene annotation process. The RNA libraries for this step were prepared from a single, whole, adult, female *P. tenuis* worm from hunter-harvested white-tailed deer from Oak Ridge, Tennessee. The nematode was collected and stored in RNAlater (Thermo Fisher Scientific Inc, Waltham, MA) at −20° C. RNA was enriched using the MasterPure (Illumina Inc, San Diego, CA) RNA Purification kit and associated protocol. After RNA extraction and purification, a transcriptomic library was prepared using the Illumina Tru-seq RNA-seq protocol. RNA was converted into cDNA using RT-PCR. Sequencing was performed with an Illumina MiSeq (Illumina Inc, San Diego, CA) at the University of Tennessee Genomics Core (Knoxville, TN). Purified RNA was loaded at 6 picomolar with 5% 6 picomolar phiX as a control on a version 3 flow cell reading 250 bases, paired end. The 20,116,257 RNA-seq reads that passed quality control were trimmed with Trimmomatric (v0.39, Bolger et al., 2014) (settings used: ILLUMINACLIP:${ADAPTERS}:4:15:7:2:true LEADING:0 TRAILING:0 SLIDINGWINDOW:4:15 MINLEN:75) and then aligned to the *P. tenuis* whole genome assembly with the splice read aligner STAR (v2.7.1a, Dobin et al., 2013) using the following settings: --alignIntronMin 10 --alignIntronMax 10000 --outFilterMultimapNmax 20. We had 16,594,862 (82.5%) reads that mapped uniquely to the genome, 1,022,828 (5.0%) reads that mapped to multiple locations, and 2,471,022 (12.3%) that were too short to map to the assembly.

Using the funannotate pipeline (v1.8.1; Palmer, 2016), we trained the *ab initio* gene prediction algorithms and annotated genes. Specifically, we performed the funnanotate train command on the RNA-seq data and genome assembly, which created a genome-guided Trinity (Grabherr et al., 2011) RNA-seq assembly and PASA (Haas et al., 2003) assembly resulting in a BAM file (Trinity), Trinity transcript file (Trinity), and a GFF3 file (PASA). These files were used in conjunction with the STAR alignments and 1,100 predicted *P. tenuis* proteins harvested from the RNA-seq data to train the *ab initio* gene predictors Augustus v3.3.3 (Stanke et al., 2006) and Genemark v4.61 (Lomsadze et al., 2014) for gene prediction with the predict command (additional options: -max_intronlen 15000). Gene models were then compared against the RNA-seq data to add untranslated regions and fix gene models that disagreed with the RNA-seq data using EvidenceModeler and PASA (Haas et al., 2008) in the funannotate update step. The output of that command is NCBI-compatible gene models, which were then used to assign functional annotations to the protein-coding gene models with the annotate command. The following databases and software were used for functional annotation: hmmer with Pfam-A database (Eddy, 2011; Mistry et al., 2020), Diamond v2.0.4.142 with UniProt DB version 2020_05 (Buchfink et al., 2021), EggNOG-mapper v1.0.3-diamond-2.0.4 with EggNOG database v.4.5 (Hyatt et al., 2010; Huerta-Cepas et al., 2016; Steinegger and Söding, 2017; Rawlings et al. 2018; Huerta-Cepas et al., 2019), SignalP v4.1 (Peterson et al., 2011), MEROPS (Rawlings et al. 2018), and CAZYme (Drula et al., 2022).

The funannotate pipeline predicted 38,371 gene models and identified 29,657 protein-coding genes (Table 1). The average gene length was 4,088 bp, with a maximum length of 128,078 bp. Using the predicted protein sequences from gene models, we assessed the proteome completeness with BUSCO and the nematode_odb10 lineage dataset. We found 71% (2,224) of the BUSCO proteins being complete and 7.1% (223) of the BUSCO proteins fragmented in our annotation. We did not detect 21.9% (684) of the BUSCO proteins.

We anticipate this *de novo* genome assembly will facilitate a broad range of studies aimed at investigating the evolution and biology of *P. tenuis* and other Protostrongylids. For example, our team has leveraged the assembly as a reference for reduced-representation methods facilitating population-level insights into the transmission of *P. tenuis*. Additionally, the annotation opens the door for genome-wide association studies, which may identify a genetic basis for pathogenicity in brainworm. This information might also be used to design vaccines or treatments to reduce morbidity and mortality in moose and other aberrant hosts.

## Database Submission

Nucleotide accession numbers associated with this announcement are SRR15359507 (BioSample SAMN20601477) for the RNA sequencing, PRJNA729714 for the whole genome assembly, and JAHQIW000000000 for the annotation.
